# Human-like interactions prompt people to take a robot’s perspective

**DOI:** 10.3389/fpsyg.2023.1190620

**Published:** 2023-10-10

**Authors:** Tian Ye, Takashi Minato, Kurima Sakai, Hidenobu Sumioka, Antonia Hamilton, Hiroshi Ishiguro

**Affiliations:** ^1^Department of Psychology, Shandong Normal University, Jinan, China; ^2^Institute of Cognitive Neuroscience, University College London, London, United Kingdom; ^3^RIKEN Information R&D and Strategy Headquarters, Guardian Robot Project, Keihanna Science City, Kyoto, Japan; ^4^Hiroshi Ishiguro Laboratories, Advanced Telecommunications Research Institute International, Keihanna Science City, Kyoto, Japan; ^5^Graduate School of Engineering Science, Osaka University, Osaka, Japan

**Keywords:** perspective-taking, human-robot interaction, humanisation, social behavior, anthropomorphism

## Abstract

The increasing need for human-robot interaction requires not only robots to understand how humans think, but also humans to understand robots. Interestingly, little attention has been given to how humans interpret robots’ behaviors. In this study, we adopted a social mental rotation task and investigated whether socially engaging behaviors could influence how people take a robot’s perspectives. In a real lab, two android robots with neutral appearance sat opposite each other by a table with conflicting perspectives. Before the participant started the experiment, one of the robots behaved more interactively than the other by showing more socially engaging behaviors. Then the participant was required to identify rotated normal or mirrored digits presented inbetween the two robots. Results revealed a significant interactive effect between the digits type (normal; mirrored) and robot type (interactive; noninteractive). When digits were oriented to the interactive robot, we found a larger RT difference between normal and mirrored digits. In general, these findings suggested that robots’ interactive behaviors could influence how people spontaneously consider the robot’s perspective. Future studies may further consider how interactive behaviors can shape human-robot relationships and facilitate human-robot interaction.

## Introduction

As more and more artificial agents come into people’s daily life, the question of how humans interact with such agents is drawing increasing attention. An important case is human-robot interaction (HRI). Many studies have tried to build robots with social intelligence, which can comprehend complex human behaviors (Johnson and Demiris, 2005; Kelley et al., 2008; Görür et al., 2017; Loghmani et al., 2017). Others have investigated people’s responses to robots by examining their explicit impressions of robot partners (Kaplan, 2004; Becker-Asano et al., 2010; Flandorfer, 2012; Mandell et al., 2017; Dubois-Sage et al., 2023). Although some studies investigated whether humans would explicitly or implicitly ascribe a mind to a robot on a perceptual level (Spatola and Wudarczyk, 2021; Li et al., 2022), limited studies have explored whether people would interact with a robot as if it has a mind. In this study, we adopted methods from social cognitive studies focused on human-human interaction, and tested whether people will spontaneously take the perspective of a robot in a way similar to how they do with another human.

Visual perspective taking (VPT) refers to the ability to consider what another person can see and how they see it (Flavell, 1977; Samson et al., 2010; Elekes et al., 2016). Previous studies on VPT suggest that people are more likely to take the perspective of an agent that they consider to be human, including agents that move like a human, look like a human, and belong to an in-group (Vaes et al., 2004; Mazzarella et al., 2012; Zhao et al., 2016; Ye et al., 2021; Zhao and Malle, 2022). Based on these findings, our previous research proposed the ‘humanisation account’ of VPT, suggesting that the psychological process of humanisation might serve as a gateway towards spontaneous VPT. The concept of humanisation originates from studies on infrahumanisation in human-human interactions, which explore how individuals attribute human-like characteristics to certain agents (Leyens et al., 2000, 2007).

In the field of Human-Robot Interaction (HRI), a closely related concept to humanisation is anthropomorphism. Numerous studies have explored the impact of anthropomorphism on HRI. However, as experiments incorporate more anthropomorphic elements, the concept of anthropomorphism becomes increasingly complex. For example, Fischer (2022) summarized anthropomorphism into three distinct meanings. First, anthropomorphism is seen as the phenomenon where people attribute human-like traits to robots, an idea closely aligned with the original definition of anthropomorphism by Epley et al. (2007), who regarded it as the act of “imbuing the imagined or real behavior of non-human agents with human-like characteristics, motivations, intentions, and emotions.” The second definition regards anthropomorphism as the human-like properties of robots, including features like facial expressions, voice, and interactivity, which is often referred to as anthropomorphic features (Rosenthal-von der Pütten and Krämer, 2014). The last perspective defines anthropomorphism as the way people react to robots as if they were human actors (Westlund et al., 2016), sometimes considered the behavioral consequences of anthropomorphism. In contrast, the psychological concept of humanisation in HRI remains closer to its original meaning and is more specific. For instance, Robert (2017) defined humanisation as “the representation of robots as humans and/or attributing human-like qualities to robots.” Spatola et al. (2021) further noted that humanisation encompasses representing artificial agents on a conceptual continuum from robots to humans. Therefore, while humans may not perceive robots as fully human, they may humanize them to a greater extent in certain contexts. To maintain conceptual clarity and consistency with our ‘humanisation account’ in the human-human VPT research, we used the concept of humanisation to describe the process by which humans attribute human-like qualities to their robot partners. We aimed to examine whether this difference in humanisation of a robot affects our perception of its mind by investigating whether humanisation could also enhance our tendency to adopt a robot’s perspective.

Existing literature indicates that the degree to which an agent is perceived as human-like influences the likelihood we engage in visual perspective taking (VPT) (Mazzarella et al., 2012; Furlanetto et al., 2013). Several studies investigated how goal-directed movements, which serve as cues indicating an agent is an intentional human agent, can elicit VPT in various contexts. Findings from these studies demonstrate that the mere presence of a human body is insufficient to trigger VPT. However, when the agent exhibits goal-directed actions, individuals tend to engage in ‘allocentric coding’ or the adoption of the agent’s perspective. For example, Mazzarella et al. (2012) presented participants with pictures showing a male actor grasping (or not) and gazing towards (or straight ahead) a bottle. They found that people have a stronger tendency to report the location of the bottle from the actor’s point of view when the actor was grasping the bottle. Similarly, Furlanetto et al. (2013) examined the influence of the disparity between grasping and gaze direction on spontaneous VPT. They replicated the findings from Mazzarella et al. (2012), further, they showed that the incongruency of grasping and gaze direction has a stronger effect in eliciting spontaneous VPT. This effect can be attributed to the increased need to infer the actor’s intention when they looked in a direction different from the one in which their hand was reaching. Overall, these studies suggest that perceiving an agent as an intentional human is a prerequisite for spontaneous VPT.

Using similar procedures, Zhao et al. (2016) investigated spontaneous visual perspective taking in HRI. In one study, they asked whether goal-directed movements could influence people taking a robot’s perspective using a number identification paradigm. Specifically, participants took part in a single trial where they were presented with a picture or a short video depicting a robot sitting on the opposite table, with the number ‘6’ displayed in the center of the table. Participants were assigned into conditions where the robot was not looking at the number (baseline condition), gazing towards (gazing condition) the number or touching the number while looking at it (gazing+reaching condition) and they were asked to report which number was on the table. The rationale is if participants spontaneously take the robot’s perspective, they would be more likely to report the number to be ‘9’ instead of ‘6’, since from the robot’s eyes, it was number ‘9’. The results indicated that when a short video was used instead of a static picture, more participants answered ‘9’ in both the ‘gazing’ and ‘gazing+reaching’ conditions. However, there was no significant difference between these conditions when participants were shown static pictures of the robot. The authors believed that presenting motions in their dynamic format increased the validity of such goal-directed behaviors, thus encouraged participants to spontaneously take the robot’s VPT.

In addition to goal-directed movements, the robot’s humanlike appearance was also found to elicit spontaneous VPT in humans. In a more recent study, Zhao and Malle (2022) systematically examined how agents with different levels of human-like appearance can prompt spontaneous visual perspective taking (VPT) differentially in individuals. They found spontaneous VPT towards robots complies with the ‘mere appearance hypothesis’, that a robot’s apparent human resemblance could trigger spontaneous VPT in humans, despite that some robots appeared eerie to humans. Corroborating the findings in both human and robot VPT studies, characteristics such as goal-directed movements and humanlike appearance might elicit the humanisation process, which could further trigger spontaneous VPT.

Humanisation can also impact VPT through top-down processes, where prior information, context or task instructions make a participant believe the agent is similar to themselves. Recent findings revealed that compared with an outgroup member, we have a stronger propensity to take an ingroup member’s perspective (Vaes et al., 2004; Simpson and Todd, 2017; Ye et al., 2021). The ingroup effect is not restricted to groups formed by physical features, but also applies to arbitrarily formed groups. Vaes et al. (2004) assigned participants to two groups by using a pseudo saliva test. They then asked participants to form ingroup or inter-group pairs and each participant needed to draw a letter ‘E’ on their foreheads. They found that in the ingroup pairs, participants tended to draw the letter to be readable from the partner’s point of view, suggesting that they were imagining seeing from the partner’s standpoint. Recently, our study directly tested the humanisation account for VPT by using a two-agent VPT task. In this study, participants wore a VR headset and were immersed in a virtual room with two agents sitting around a table. The two agents sat opposite each other, thus they have conflicting views of items presented in the centre of the table. In each trial, participants needed to report whether a letter presented on the table is normal or mirror-reversed. As the letter was oriented towards one of the agents, if participants have a stronger propensity to take one of the agents’ perspective, they should have better task performance on items oriented to that perspective (Ye et al., 2021). By using this paradigm, researchers showed that participants had a stronger propensity to take the ingroup agent’s perspective (vs. outgroup perspective), and a moving agent’s perspective (vs. a static agent’s perspective). This study provided direct evidence supporting the humanisation account for VPT.

Despite these current findings, there is still more to investigate in relation to the humanisation account of VPT. In the research reviewed above, there are at least three factors which can make people humanise an agent and consider the agent’s perspective (1) the human-like appearance of the agent; (2) the goal-oriented behavior of the agent; (3) contextual information about ingroup membership. Here, we test whether a different factor can also induce humanisation: socially engaging behavior. Socially engaging behaviors, such as saying hello or waving to a person, are often used at the start of interaction and can set up how people engage with each other (Eriksson, 2019; Holler, 2022; Hamilton et al., 2023). Here, we test if socially engaging behavior from a robot can induce humanisation of the robot and enhance people’s propensity to spontaneously take the perspective of the robot. Above all, humanlike behavior is an important aspect pursued by robot designers and has become more and more natural recently, which makes it an effective way to convey information that can induce individuals to humanise a robot. Using real robots instead of their pictures in the real world, we can create nonhuman agents with less or more humanlike features, and examine if humanisation is also a prerequisite for spontaneous VPT in human-robot interaction.

**Figure 1:**
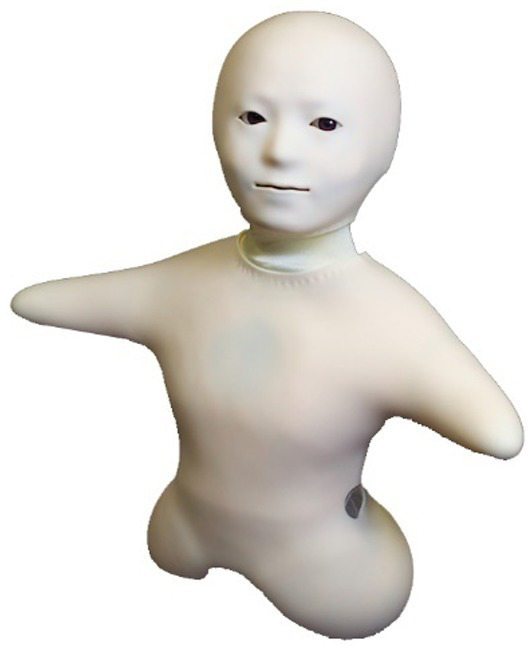
A visual representation of the Telenoid robot during the experiment. During the experiment, two Telenoid robots were positioned, each supported by an individual tripod. For a detailed layout of the experiment, please refer to Figure 2.

In our current study, we employed a social mental rotation paradigm to directly investigate whether individuals are more inclined to adopt the perspective of a robot when it exhibits more human-like behaviors in real-world scenarios. Notably, in previous HRI studies, researchers have often focused on one-to-one interaction. However, in the current study, we asked participants to directly interact with two robots simultaneously. In everyday life, people are frequently exposed to multiple perspectives. Although cognitive studies have seldom tested how individuals interact with more than one agent at a time, being exposed to multiple perspectives simultaneously requires us to select which perspective to adopt since our cognitive system operates with limited resources. Compared with the one-to-one interaction, the one-to-many interaction can also possibly induce self-focused attention, which may thereby reduce VPT, as suggested by a study on social network sites (Chiou and Lee, 2013) Our previous study has shown that perspective selection is an effective way to study VPT in virtual reality (Ye et al., 2021). Thus, consistent with our previous study, we used two Telenoid robots in the current study (Ogawa et al., 2011, see Figure 1), allowing participants to directly compare their differences in socially engaging behaviors. We specifically chose the Telenoid robots due to their identical and neutral appearances, minimizing the potential influence of mnemonic associations or extreme attitudes on participants’ responses. By “neutral” we mean two things in this context. Firstly, it refers to Telenoid having a moderate level of human resemblance. Telenoid robot scored 53.13 on the ABOT human-likeness scale (with 0 indicating the robot is “not-human at all” and 100 indicating “just like a human,” see Phillips et al., 2018; Kim et al., 2022), which means people represent it in the middle on the robot-human continuum. Secondly, Telenoid is considered to have a neutral gender. In the ROBO-GAP database, Telenoid scored 4.48 on a 7-point scale, indicating that people have difficulty associating it with either the female or male category (Perugia et al., 2022). It is also worth mentioning that the perceived age of Telenoid is 26.4 on average, with a standard deviation of 19. The relatively large SD suggests that people find it challenging to relate it to a specific age group (Perugia et al., 2022).

We adopted a 2 (Robot-type: interactive, noninteractive) by 2 (Digit-type: normal, mirror-reversed) within-subject design. At the beginning of the experiment, one of the robots interacted with the participant in a humanlike way for a short period, then participants conducted the social mental rotation task which serves as a measurement of spontaneous VPT. At each trial, a normal or mirror-reversed digit would appear at the center of the table oriented at a specific angle. Participants were instructed to identify whether it was normal or mirror-reversed as quickly and accurately as possible. We hypothesise that the simple interactive activities ahead of the mental rotation task would result in participants humanising the two robots differently, and they would engage in a stronger propensity to process items oriented towards the interactive robot. Thus, items facing the interactive robot might be recognized faster or more accurately. To guarantee the reliability of our results, we pre-registered all the data analysis procedures with Open Science Framework.^1^

## Method

### Participants

Our recruitment target for this study was 36 participants, and we tested 44 participants with the expectation that some datasets might be excluded due to different data exclusion criteria according to our pre-registered document (see Footnote 1). This sample size was based on informal comparisons to Ye et al. (2021) which used a similar experimental design in a VR setting in the UK, where a power analysis showed *N* = 36 is appropriate for a repeated measures ANOVA with an expected effect size of 0.25 and 80% of power. Note this power analysis was not part of our formal pre-registration but the target sample size was. All the participants are right-handed adults, with normal or corrected-to-normal vision and no recent injury to their right-hand index and middle fingers. Participants were recruited from commercially available lists in Japan provided by a commercial company and from a local commercial recruiting website. Participants received their payment based on a 1,500 JPY per hour rate to compensate for their time for the experiment, plus extra money to cover their transportation fee. This study was approved both by the UCL ethics research committee and follows the Declaration of Helsinki 1964, and by the ATR ethics committee for studies involving human participants.

### Materials, stimuli, and design

Two Telenoid robots (named Nitro and Zeto) were used as the agents in our study. Telenoid is a teleoperated android robot. To give it a neutral appearance, its skin is white soft vinyl and it only has two eyes, a nose and a mouth which are necessary parts for communication, but endow minimal human likeliness. Its head can rotate between −40 to 40 degrees on the yaw axis, −40 to 40 degrees on the pitch axis, and − 40 to 40 degrees on the roll axis and its two short arms can move up to 40 degrees. Its eyes can move 30 degrees in the yaw axis and 30 degrees in the pitch axis. These features enable it to symbolize several social behaviors such as nodding or waving. In the current experiment, we programmed four movements for the two Telenoids to convey interactive information when necessary. They are:

1. Looking straight: the robot looks straight to the front;
2. Head turn: the robot looks at the side towards the participants at a 50-degree angle (both neck and eyeball move together), which is a sign showing it noticed the arrival of the participant;
3. Handwaving: the robot waves its hands three times showing it welcomes the arrival of the participant;
4. Looking down at the table: the robot looks down 45 degrees with neck and eyeball movements to gaze towards the centre of the table.

**Figure 2:**
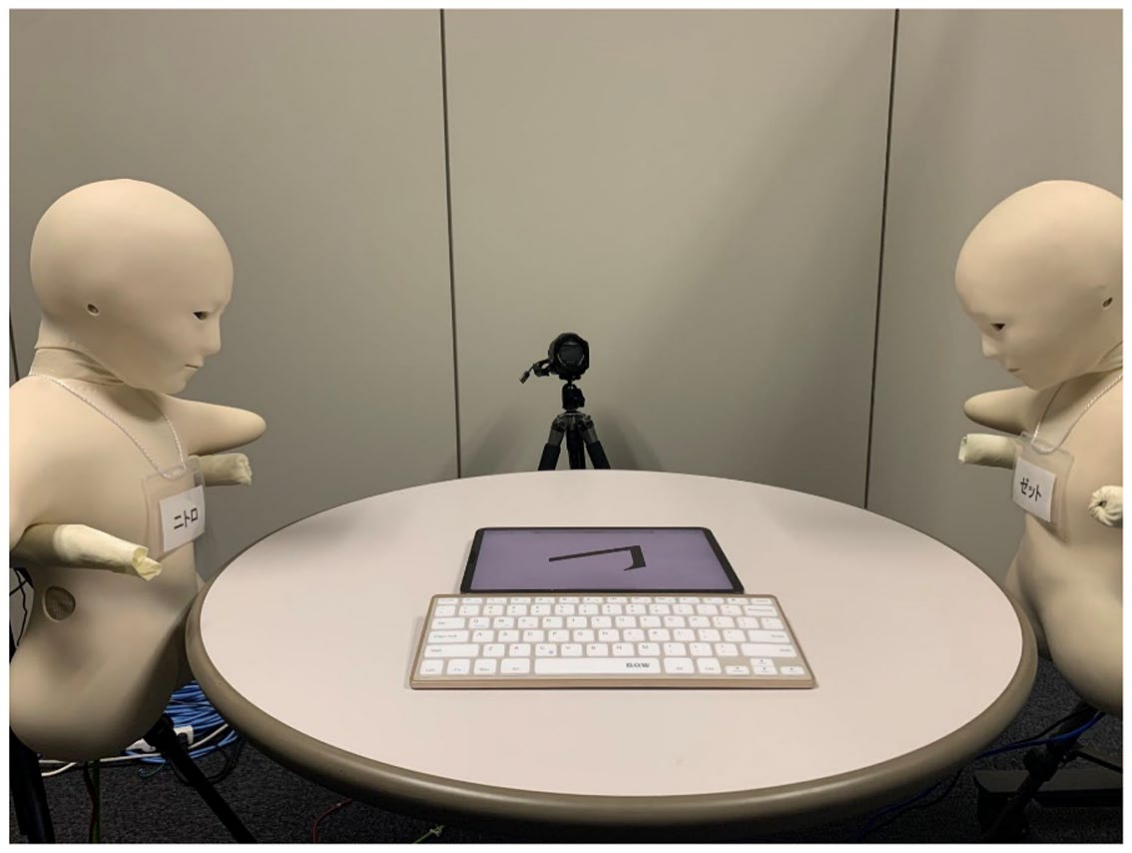
An experiment demonstration from the participant’s viewpoint. While the participant was completing the number-verification task, the two Telenoid robots only exhibited slight movements such as ‘breathing’.

The two robots were placed on the left and right side of a round table at a distance of about 10 cm to the table. To make sure participants can distinguish between them, both of the robots wore a name tag during the task. The name tags and sitting positions of the interactive/noninteractive robots were counterbalanced across all participants. In our current task, we used a ‘Wizard of Oz’ approach. During the experiment, one of the experimenters controlled the robots from a separate room, with the control room separated from the laboratory by an opaque partition wall. The experimenter observed participants through a webcam, keeping them unaware that a human operator was controlling the robot’s actions. When the participant was guided to sit on the chair, the interactive robot would look towards the participant and then wave its hands, while the noninteractive robot looked down at the table. Then the interactive robot turned its head and looks straight to the front. While the experimenter was introducing the two robots to the participants, the interactive robot turned its head to the participants again when its name was called by the experimenter, and waved its hands again. Right before the task begins, both robots looked down towards the table. During the experiment, both robots displayed only slight movements such as ‘breathing’ (Figure 2).

Four asymmetric Arabic digits (2, 4, 5, 7) in Times New Roman font were used for the social mental rotation task. Digits were displayed by using the iPad Pro (2018) which was placed flat on the table in front of the participant and between the robots, appearing in an invisible square with a size of ~16*27 degree visual angle viewing from a ~ 50 cm distance. Participants were instructed to use a Bluetooth keyboard (B.O.W keyboard) to make keypresses. Each participant completed 64 trials, with 24 trials displaying digits towards the left, 24 trials to the right and 16 trials presenting digits to the participants. In each orientation of the digits, the number of trials of the normal and mirrored digits is the same, and the number of trials of each digit (2, 4, 5, 7) is also the same under both normal and mirrored conditions. It is worth noting that additional egocentric perspective trials were incorporated to introduce uncertainty regarding the orientation of the digits and to mask the true purpose of the study. However, the results from these egocentric perspective trials were not included in the statistical analyses. All types of trials were fully randomized in the experiment. Participants used the left/right arrows on the keyboard to respond, by pressing the left arrow when they saw a normal digit and the right arrow if the digit was mirror-reversed.

To measure participants’ subjective impression of the two robots, a post-experiment questionnaire was administered, consisting of three parts. The first part of the questionnaire is about the impression of Nitro, which is composed of the IMI relatedness subscale (McAuley et al., 1989), the Group Identification questionnaire (Doosje et al., 1995; Hertel and Kerr, 2001), and the Godspeed Questionnaire Series (Bartneck et al., 2009). The IMI relatedness subscale included eight items, which was commonly used when participants involved in interpersonal interactions (Huygelier et al., 2019; Taub et al., 2020, visiting https://selfdeterminationtheory.org/intrinsic-motivation-inventory to access the relatedness subscale of this inventory). A typical statement of the IMI relatedness subscale was ‘I’d like a chance to interact with Nitro more often’. The Group Identification Questionnaire was a collection of four statements and was included to measure participants’ identification towards the two robots (e.g., ‘I see myself as a member in the group which Nitro belongs’). The Godspeed Questionnaire Series had 24 bipolar scales, and was usually used to measure participants’ perceptions of robots during HRI. The second part of the questionnaire was identical to the first part, but targeting at their impression of Zeto. Finally, the last part of the questionnaire was composed of four self-generated questions, which focused on participants’ subjective feelings during the task, and their evaluation of the difficulty of the task (e.g., ‘When I was doing the experiment, I feel very relaxed’). For the Group Identification Questionnaire, the IMI and questions in part 3, participants answered each question on a 7-point Likert scale. Each trial came with a statement, and participants then selected a number between 1 (totally disagree) to 7 (totally agree) to indicate how much they agreed with the statement. For the Godspeed questionnaire, participants needed to rate on a 5-point semantic differential scale between a pair of antonyms, to indicate the extent to which they associate the specific attribute to the robot (e.g., ‘inert/interactive’).

### Procedure

Participants arrived at the lab and signed the consent forms first. Then they were given a verbal instruction for the experiment, with a printed version of the instruction. After the introduction, participants needed to complete a practice of about 10 trials, to make sure they understand the requirement of the task.

Then they were guided to the formal experiment room and introduced to the two robots. The other experimenter monitored the room via a web camera, and upon the arrival of the participant, she would control the two robots to make one of them turn to look at the participant and wave hands, while the other looked down towards the table. Participants were instructed to sit in front of a round table, and the experimenter would then introduce the robot’s names to the participant. When called its name, the interactive robot would look at the participant and wave its hand again. If the participant asked what things the robot can do, the experimenter would say they can see and move. Then the participant starts the social mental rotation task.

The script is programmed using Java (v12.24.1). Each trial began with a ‘+’ focus presented for 300 ms, then a normal or mirror-reversed digit would be displayed until the participant presses a key, or until 3,000 ms if no keypress was detected (Figure 2). Participants were instructed to make a response as fast and as accurately as possible. Then the next trial would start after 500 ~ 1,000 ms. Participants needed to complete 64 trials. The whole social mental rotation task lasted for about 3 ~ 5 min.

After the social mental rotation task, participants filled out the post-experiment questionnaires on a laptop. Questions were presented by using Matlab PsychToolbox 3, and only one question was displayed each time. Participants chose a number by clicking on the rating scale. There was no time limitation for participants to answer each question.

## Results

### Data analysis

Consistent with our previous study (Ye et al., 2021), we analyzed both the accuracy and the reaction time data for each participant. According to the criteria we pre-registered (see Footnote 1), datasets that had overall accuracy or mean RTs beyond 2 SD were excluded. For each participant, trials with an RT less than 150 ms or beyond 3 SD were excluded from analyses. Following these criteria, datasets from three participants were excluded from further analysis. Two of them were because of low overall accuracy and one was because of long RT. In the end, 41 datasets were included in the final analyses (22 males, 26.1 ± 6.3 years old). Since our previous study showed that both the type of the item and the orientation of the item could influence a participant’s performance (Ye et al., 2021), we applied a 2 (Robot: interactive; non-interactive) × 2 (Digit-type: normal; mirrored) repeated-measurement ANOVA analysis. We relied on measurements of accuracy and RT, but also conducted exploratory analyses with Inverse Efficiency Scores.

In addition, we also tested if participants’ performance on the social mental rotation task was correlated with their subjective reports on the questionnaires. To do this, we calculated the differential score by subtracting the participant’s overall accuracy or mean RT in the ‘noninteractive robot’ condition from those in the ‘interactive robot’ condition, and conducted Pearson correlation tests to obtain their correlational coefficients with the differential scores on different questionnaires.

### Accuracy

**Figure 3:**
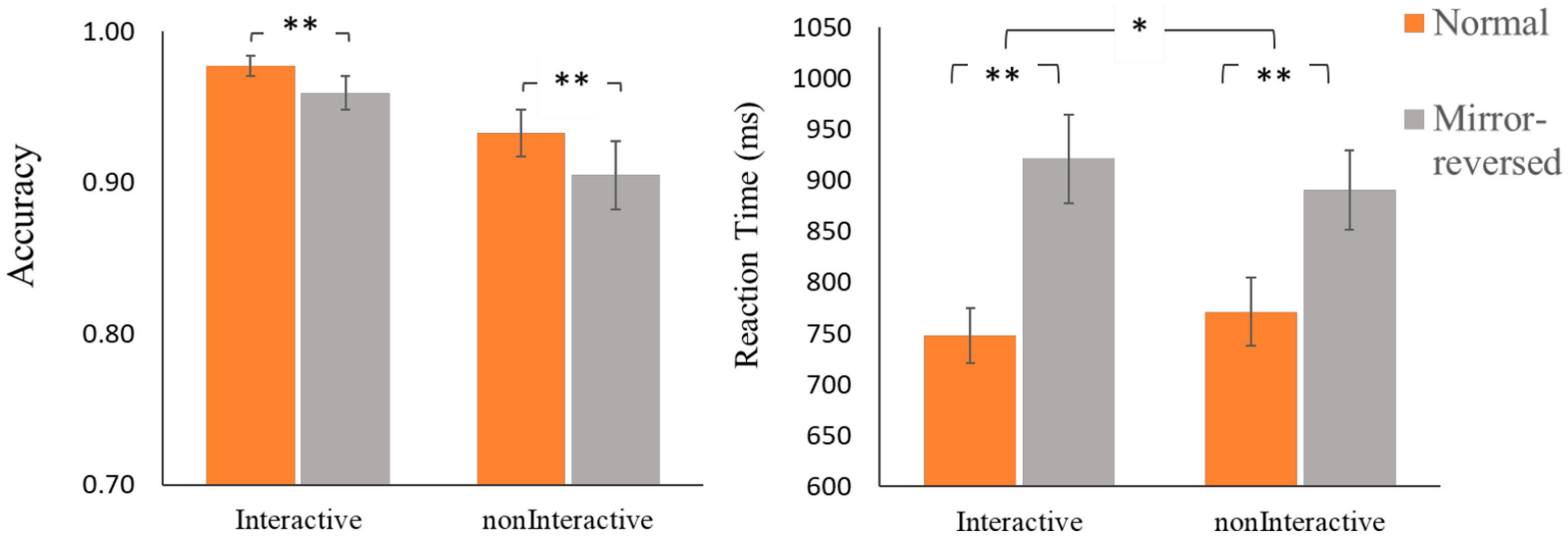
The accuracy and reaction time analysis results (^*^: *p* < 0.05; ^**^: *p* < 0.01).

The two-way repeated measurement ANOVA with Digit-type and Robot-type both as within-subject factors revealed a significant main effect for Digit-type: *F* (1, 40) = 8.98, *p* = 0.005, η*_p_*^2^ = 0.18, but the main effect for Robot (*F* [1, 40] = 2.58, *p* = 0.116) and the interaction effect (*F* [1, 40] = 0.14, *p* = 0.712) were not significant (see Figure 3).

### Reaction time

The two-way repeated measurement ANOVA with Digit-type and Robot-type both as within-subject factors revealed a significant main effect for Digit-type: *F* (1, 40) = 39.88, *p* < 0.001, η*_p_^2^ = 0.50*, but the main effect for Robot was not significant (*F* [1, 40] = 0.07, *p* = 0.793). Critically, results showed a significant interaction effect between the two factors (*F* [1, 40] = 5.03, *p* = 0.031, η*_p_^2^ = 0.112,* Figure 3). Nevertheless, paired-sample *t*-tests indicated no significant difference between conditions where normal digits were oriented towards the interactive and noninteractive robot (*t* [40] = −1.56, *p* = 0.127); or between conditions for mirror-reversed digits (*t* [40] = 1.50, *p* = 0.142). Thus, the significant interaction effect might be the result of a relatively larger difference in RT between normal and mirror-reversed digits for the interactive robot compared to the noninteractive robot.

### Inverse Efficient Score (IES)

We repeated the analysis procedures of accuracy and RT on the IES considering that the speed-accuracy trade-off might influence our results. Only the main effect of Digit-type reached the significant level (*F* (1, 40) = 82.77, *p* < 0.001, η*_p_^2^ = 0.674*). Neither the main effect of Robot-type (*F* [1, 40] = 1.18, *p* = 0.285) nor the interaction of these two factors (*F* [1, 40] = 0.328, *p* = 0.570) was significant.

### Questionnaire results

On all three questionnaires used in our study, participants gave higher ratings to the interactive robot than the noninteractive one (Intrinsic Motivation Inventory: *t* (40) = 2.42, *p* = 0.02, cohen’s *d* = 0.41; Group Identification Questionnaire: *t* (40) = 3.06, *p* = 0.004, cohen’s *d* = 0.49; Godspeed Questionnaire: *t* (40) = 3.78, *p* = 0.001, cohen’s *d* = 0.70). For the descriptive results on the three questionnaires see Table 1.

**Table 1:** Descriptive results for the Intrinsic Motivation Inventory, Group Identification Questionnaire and Godspeed Questionnaire.

	Interactive robot		Noninteractive robot	
	Mean	SD	Mean	SD
Intrinsic Motivation Inventory	35.85	8.03	32.80	6.70
Group Identification Questionnaire	13.27	5.17	11.05	3.79
Godspeed Questionnaire	70.46	16.32	60.51	11.65

### Correlation results

To test the hypothesis that participants’ different subjective feelings towards the interactive and noninteractive robot are correlated with their task performance on the social mental rotation task, we calculated the differential scores and then tested their intercorrelations. The differential scores were calculated for each index by subtracting participants’ ratings (or behavioral indices) of the noninteractive robot from that of the interactive robot. For example, for the Intrinsic Motivation Inventory (IMI):

IMI differential score = IMI score of the interactive robot – IMI score of the noninteractive robot.

Each differential score was then entered into a correlation matrix to calculate their correlational coefficients. The correlational matrix was designed in Table 2. Results revealed that the correlation between the IMI differential score and RT differential score for normal digits is significant (*r* = 0.315, *p* = 0.045), suggesting that when participants had a stronger motivation to interact with the interactive robot, their responses for normal digits oriented towards the interactive robots were slower. However, this effect would not survive a correction for multiple comparisons (with Bonferroni correction).

**Table 2:** The results of Pearson correlational analyses.

		Intrisinc Motivation Inventory (8 items)	Group Identification Scale	Godspeed Questionnaire	Overall Accuracy	Accuracy: Normal Number	Accuracy: Mirrored Number	Mean RT	RT: Normal Number
		Inter - nonInter	Inter - nonInter	Inter - nonInter	Inter - nonInter	Inter - nonInter: normal	Inter - nonInter: mirrored	Inter - nonInter	Inter - nonInter: normal
Group Identification	Inter-nonInter	0.589^**^							
Godspeed Questionnaire	Inter-nonInter	0.615^**^	0.712^**^						
Overall Accuracy	Inter-nonInter	−0.078	0.148	0.027					
Accuracy: Normal Number	Inter-nonInter: normal	−0.033	0.051	−0.026	0.391^*^				
Accuracy: Mirrored Number	Inter-nonInter: mirrored	−0.078	0.146	0.045	0.933^**^	0.035			
Mean RT	Inter-nonInter	0.116	−0.118	−0.016	−0.186	−0.239	−0.123		
RT: Normal Number	Inter-nonInter: normal	0.315*	0.058	0.192	−0.208	−0.137	−0.187	0.644^**^	
RT: Mirrored Number	Inter-nonInter: mirrored	−0.082	−0.196	−0.163	−0.088	−0.209	−0.021	0.825^**^	0.099

The matrix contains correlational coefficients. (^*^: *p* < 0.05; ^**^: *p* < 0.01).

## Discussion

This study investigated whether people would be more likely to take the perspective of a more humanised robot. We implemented a social mental rotation task, where participants needed to identify normal or mirror-reversed digits while two Telenoid robots were also looking at the same stimuli. At each trial a digit would randomly be oriented to one of the robots or the participant. We hypothesized that participants would respond either more quickly or more accurately to digits oriented to the more interactive robot’s perspective. Interestingly, our pre-registered analyses showed that when digits were oriented towards the interactive robot, there was a larger difference in RT between normal and mirrored digits. The correlational analyses also revealed a weak result that participants’ intrinsic motivation to interact with the two robots might influence their processing of the robot’s perspectives. As such correlational result could not survive multiple comparisons, we decided not to discuss it in detail.

Our initial hypothesis posited that individuals would display a stronger inclination to adopt the perspective of the interactive robot, which would be reflected in faster and more accurate performance when the digits were oriented towards that particular robot. Our results partially supported this hypothesis. A brief interaction prior to the experiment indeed resulted in participants forming distinct impressions of the two robots. Irrespective of their names or sitting positions, participants consistently gave the interactive robot higher ratings across all three questionnaires. We believe that this impression disparity further contributed to slight differences in cognitive processing, as we observed a larger RT difference between normal and mirror-reversed digits when they oriented towards the interactive robot. This intriguing result can be interpreted in two ways. First, this result could be regarded as a reflection that participants had a weak tendency to consider a more interactive robot’s perspective, probably due to the relatively brief duration of the greeting period in the current experiment. With only two instances of socially engaging behaviors exhibited by the interactive robot (during the initial introduction and when its name was called), it is possible that as participants grew more acquainted with the robots, their impressions of them might have further diverged, potentially leading to greater variations in their propensity to adopt the robots’ perspectives. Second, it is plausible that how participants take a nonhuman agent’s perspective depends on the complexity of the visual input, i.e., taking a robot’s perspective depends on the task difficulty. It is possible that the identification of normal and mirror-reversed digits is indeed accomplished by two independent systems: identifying a normal digit is relatively easy and can be completed by System 1, which is fast and effortless; whereas identifying mirror-reversed digits entails deliberate cognitive thinking and might be completed by System 2, which is flexible but cognitively demanding (Stanovich and West, 2000; Kahneman, 2003). Hence, any advantage in response times stemming from the initial propensity to adopt the interactive robot’s perspective might be overshadowed by the more substantial individual differences in deliberately processing the mirror-reversed digits. While the present experimental design does not offer conclusive data to examine these two hypotheses, prior studies have demonstrated that individuals perceive different “minds” from various robots during human-robot interaction, and attribute distinct tasks to emotionally capable or incapable robots (Wiese et al., 2022), or ascribe the robot’s ‘beliefs’ differently (Thellman and Ziemke, 2020). Future studies may delve into whether task difficulty influences individuals’ attribution of minds to robot-like agents.

Our current results stressed the importance of social behaviors in HRIs, and more particularly the importance of socially engaging behaviors. In the current study, we used two Telenoid robots which tend to be perceived neutral in terms of human-likeness and gender. By having such control over the robots’ characteristics, we were able to better elucidate the distinct role of socially engaging behaviors in eliciting spontaneous perspective-taking (VPT). Although many previous studies have revealed that some social behaviors, such as gazing or reaching, can affect how humans consider a robot’s mind, they mainly focused on goal-directed behavior. For example, in the study from Zhao and Malle (2022), researchers found that rather than following an ‘uncanny valley’ pattern, participants tended to take the perspective of a robot as it appeared more like a human. Critically, they revealed that participants were more likely to take the robot’s perspective when they displayed ‘reaching’ or ‘gazing’ behaviors regardless of its appearance. These findings align with earlier research that demonstrated how goal-directed behaviors, like “gazing” or “pointing,” prompt individuals to attribute mental states to robots (Salem et al., 2011; Takahashi et al., 2013; Abubshait and Wiese, 2017). Indeed, even for young children, goal-directed behavior such as ‘gazing’ can trigger them to think about the robot’s mind thus imitating a robot’s unconsummated behaviors (Itakura et al., 2008). Our results contribute to this body of research by emphasizing the importance of socially engaging behaviors, such as greetings and establishing eye contact, in attributing mental states to robots. Compared with other forms of social behaviors, such simple forms of social behaviors normally appear at the beginning of human-human interactions, and can impact on human’s first impression when introduced to a robot during HRI. Although in Zhao and Malle (2022) study they did not find a significant interaction effect between goal-directed behaviors and humanlike appearance in impacting on taking a robot’s perspective, it would be interesting to test whether socially engaging behaviors provide additional benefits for more humanlike robots in HRI.

It is also worth noting that in the current study participants interact with two robots simultaneously. Although this one-to-many manipulation is more ecologically valid, this may limit us in comparing the current results with those in previous studies, as the majority of them investigated HRI in a one-to-one setting. It could be possible that when exposed to one robot, socially engaging behaviors may generate a larger influence on VPT, since exposing to only one robot allows individuals to concentrate on and examine its appearance, identity, as well as its socially engaging behaviors more comprehensively.

In line with our previous research (Ye et al., 2021), the current study adds evidence to the ‘humanisation account’ in social cognition. As social animals, many times we exhibit typical or standard behaviors in social contexts, such as waving to our friends, focusing on our partner during a conversation and nodding to show our agreement. Such simple social behaviors from a partner provide social cues that we are interacting with a capable social member, and elicit us humanising them to explain or predict their actions. Such behaviors are so connected with their social meanings, hence people are accustomed to using them and sometimes even generalize their meanings to agents who do not have a mind (Higgins, 1992). Our results suggest that such a ‘humanisation account’ may also hold for HRI, as a brief encounter with an interactive/noninteractive robot could induce us hold distinct attitudes towards them and thus may involve their perspectives differently. Although previous studies have shown that people in general have a reduced tendency in processing nonhuman agent’s mental states (Zhao et al., 2016), future research may consider testing other types of robot behavior and how this can impact humanisation and mindreading. It is possible that the more we perceive, or believe that the other agent can see, think or behave, the more likely we would ascribe their mind during our interaction (Bardi et al., 2019). Thus humanisation might also become a stronger account as an individual’s exposure to robots increases.

## Data availability statement

The original contributions presented in the study are included in the article/supplementary material, further inquiries can be directed to the corresponding authors.

## Ethics statement

The studies involving humans were approved by the UCL ethics research committee and the ATR ethics committee for studies involving human participants. The studies were conducted in accordance with the local legislation and institutional requirements. Written informed consent for participation in this study was provided by the participants’ legal guardians/next of kin. Written informed consent was obtained from the individual(s) for the publication of any potentially identifiable images or data included in this article.

## Author contributions

TY, TM, HS, AH, and HI designed the experiment and revised the manuscript. KS programmed the robots’ movements. TY collected experiment data and wrote the manuscript. All authors contributed to the article and approved the submitted version.

## Footnotes

^1^ https://osf.io/bqaju/
